# Patch cloning method for multiple site-directed and saturation mutagenesis

**DOI:** 10.1186/1472-6750-13-91

**Published:** 2013-10-29

**Authors:** Naohiro Taniguchi, Sayumi Nakayama, Takashi Kawakami, Hiroshi Murakami

**Affiliations:** 1 Department of Life Sciences, Graduate School of Arts and Sciences, The University of Tokyo, 3-8-1 Komaba, Meguro-ku, Tokyo 153-8902, Japan

## Abstract

**Background:**

Various DNA manipulation methods have been developed to prepare mutant genes for protein engineering. However, development of more efficient and convenient method is still demanded. Homologous DNA assembly methods, which do not depend on restriction enzymes, have been used as convenient tools for cloning and have been applied to site-directed mutagenesis recently. This study describes an optimized homologous DNA assembly method, termed as multiple patch cloning (MUPAC), for multiple site-directed and saturation mutagenesis.

**Results:**

To demonstrate MUPAC, we introduced five back mutations to a mutant green fluorescent protein (GFPuv) with five deleterious mutations at specific sites and transformed *Escherichia coli* (*E. coli*) with the plasmids obtained. We observed that the over 90% of resulting colonies possessed the plasmids containing the reverted GFPuv gene and exhibited fluorescence. We extended the test to introduce up to nine mutations in Moloney Murine Leukemia Virus reverse transcriptase (M-MLV RT) by assembling 11 DNA fragments using MUPAC. Analysis of the cloned plasmid by electrophoresis and DNA sequencing revealed that approximately 30% of colonies had the objective mutant M-MLV RT gene. Furthermore, we also utilized this method to prepare a library of mutant GFPuv genes containing saturation mutations at five specific sites, and we found that MUPAC successfully introduced NNK codons at all five sites, whereas other site remained intact.

**Conclusions:**

MUPAC could efficiently introduce various mutations at multiple specific sites within a gene. Furthermore, it could facilitate the preparation of experimental gene materials important to molecular and synthetic biology research.

## Background

Site-directed and saturation mutagenesis are widely used to prepare mutant genes for studying a protein function in basic research and for preparation of engineered proteins for industrial and pharmaceutical applications. For example, protein function is studied by comparing the wild-type protein with the corresponding mutant proteins produced from genes with single or multiple mutations. Site-directed saturation mutagenesis combined with directed evolution strategies were also used to modify protein properties [1-4], such as altering substrate specificity [5-7], enhancing activity [8-11], or improving thermal stability [12,13]. To facilitate the preparation of mutant genes, various methods of multiple site-directed and saturation mutagenesis have been developed.

The most commonly used method for site-directed mutagenesis is the QuikChange^®^ method (Stratagene, La Jolla, CA, USA), which employs mutagenic plasmid amplification to introduce single site-directed mutations. Using a more complicated protocol, this method was extended to introduce up to five site-directed mutations, although the complete mutagenesis efficiency decreased to 32% [14]. To improve this efficiency, OmniChange was developed and was used to successfully introduce five site-directed saturation mutations [15]. Although this method demonstrated high efficiency for complete multiple mutagenesis, synthesis of phosphorothioate nucleotides is necessary to generate 3′-overhanging DNA fragments.

Since 1990, several homologous DNA assembly methods [16-21] have been developed; one method in particular, one-step isothermal *in vitro* recombination (the ISO method) [20], was used to introduce multiple site-directed mutations [22]. In this method, termed as multichange isothermal *in vitro* recombination (the MISO method), multiple DNA fragments were first prepared using polymerase chain reaction (PCR) followed by gel purification. The DNA fragments possess 40-base pair (bp) sequences homologous to the adjacent DNA fragments on either side. Because the ISO reaction employs T5 exonuclease, DNA fragments are degraded in a 5′–3′ direction, exposing a 3′ overhanging sequence complementary to the adjacent DNA fragment. The DNA fragments are annealed, and the gaps are filled using Phusion DNA polymerase, and the resulting nicks are sealed by *Taq* DNA ligase. This method permits highly efficient assembly of up to six DNA fragments and is used to successfully introduce eight point mutations in a gene. However, because the ISO method was originally generated to assemble DNA fragments several hundred kilobases in length, the reaction condition was not necessary suitable for multiple site-directed mutagenesis.

**Figure 1:**
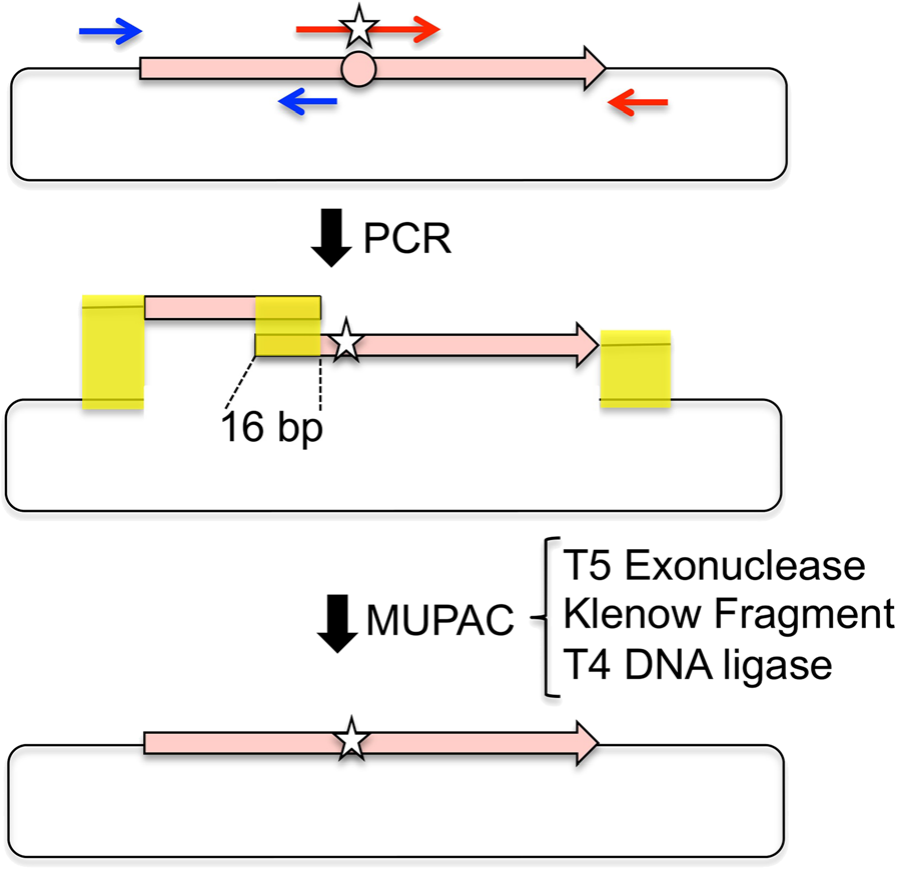
**Schematic illustration of the multiple patch cloning procedure.** DNA fragments are amplified by polymerase chain reaction using two sets of oligo-DNA primers (shown in red and blue). The star on the primer indicates the site of mismatch. The resultant DNA fragments and digested vector DNA containing 16 bp homologous regions (shown in yellow) were assembled at 37°C by T5 exonuclease, Klenow fragment and T4 DNA ligase.

In this study, we developed an optimized DNA assembly method, termed multiple patch cloning (MUPAC), which could assemble up to 11 DNA fragments to introduce nine site-directed mutations (Figure 1). The MUPAC reaction mixture is easily prepared from commercially available common enzymes, including T5 exonuclease, Klenow fragment (exo-), and T4 DNA ligase. We also utilized MUPAC to introduce site-directed saturation mutations at multiple sites in GFPuv gene. Because MUPAC is a cost effective and efficient method for the DNA manipulation, this method would be a useful tool to prepare various mutant genes to study and improve protein’s functions.

## Results

### Multiple patch cloning method

MUPAC is an optimized mutagenesis/cloning method that allows the assembly of multiple dsDNA using short 16-bp assembly sequences (Figure 1). MUPAC employs T5 exonuclease, Klenow fragment (exo-), and T4 DNA ligase. In the reaction mixture, T5 exonuclease degraded the DNA in a 5′–3′ direction and exposed 3′ overhang in the assembly regions at the ends of each fragment. When the DNA fragments annealed at the exposed complementary single-strand region, Klenow fragment (exo–) filled the gaps and T4 DNA ligase joined the nick to assemble dsDNA. The difference between MUPAC and the ISO method was the enzymes that were used in the reaction mixture. Because the ISO method employed thermostable DNA polymerase (Phusion DNA polymerase) and DNA ligase (*Taq* DNA ligase) instead of the Klenow fragment (exo-) and T4 DNA ligase, the optimum temperature for the ISO reaction was 50°C [20,22], which favored a long assembly sequence, typically 40 bp. When the method was applied to a multiple site-directed mutagenesis in MISO, this feature became drawback because the preparation of relatively long oligo-DNAs was required to introduce each point mutation. In contrast, MUPAC required an assembly sequence of only 16 bp because the optimal reaction temperature for the enzymes used was 37°C. This difference also made it easy to prepare a saturation mutant gene pool using short oligonucleotides as mentioned in the section below.

### GFPuv single site-directed mutagenesis

**Figure 2:**
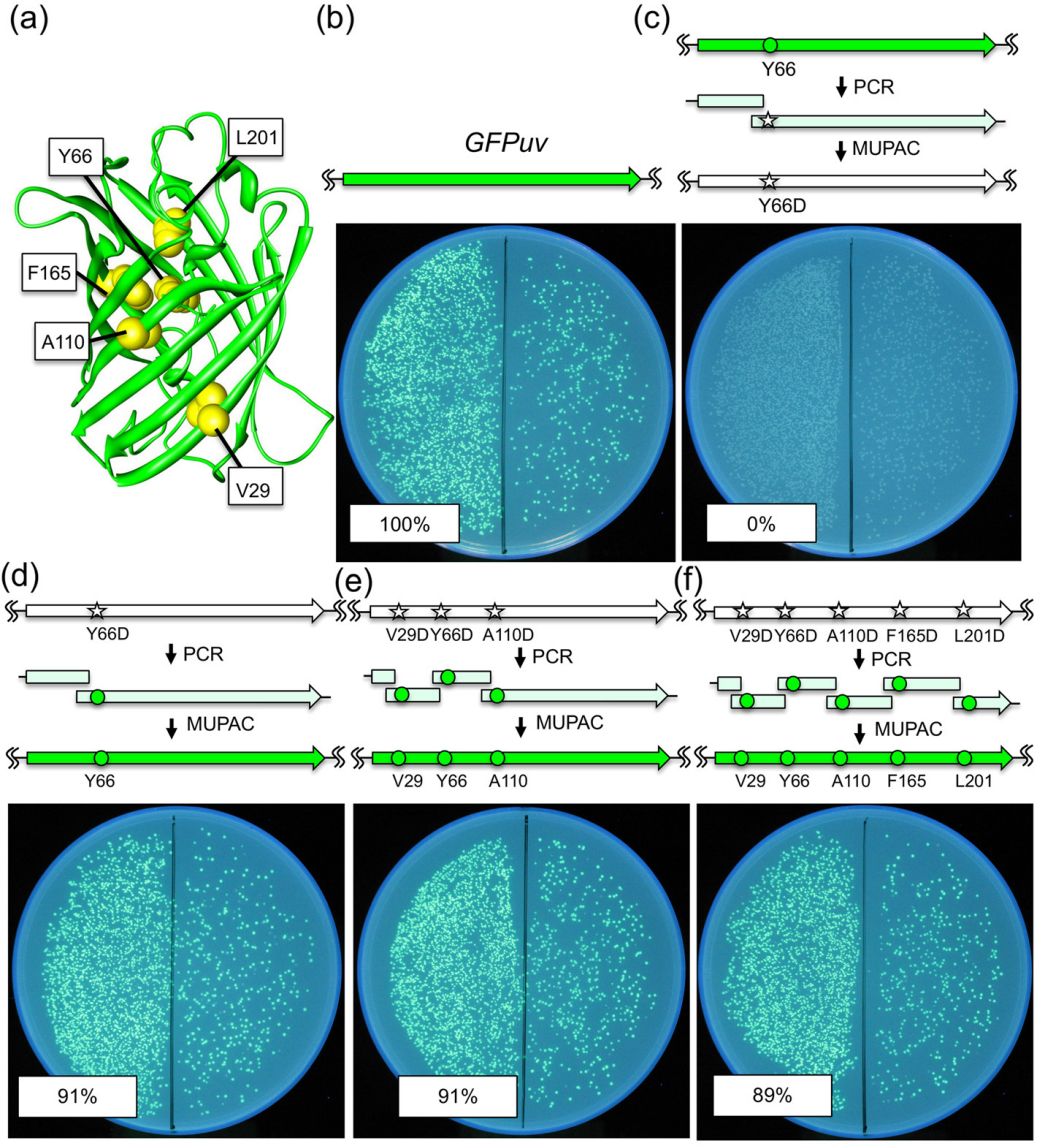
**Multiple site-directed mutagenesis of GFPuv. (a)** Three-dimensional structure of GFP (PDB ID: 1GFL) depicted using the ribbon model. Molecular graphics was created with the program UCSF Chimera (http://www.cgl.ucsf.edu/chimera/). The five residues, indicated by yellow balls, were selected as mutation sites because each was essential for GFPuv fluorescence. **(b)** Wild-type GFPuv in the pBAD vector was used to transform *E. coli* JM109. The tenth aliquot of each transformant was spread on the right half of an LB agar plate, and the remainder on the left half. The percentage frequency of fluorescent colonies is indicated at the bottom left. **(c)** A point mutation (Y66D) was introduced in GFPuv by MUPAC. One **(d)**, three **(e)**, or five **(f)** pre-introduced mutations were simultaneously restored to wild type by MUPAC. CFU in each experiment are listed in Additional file 1: Table S3.

To evaluate the performance of new enzyme mixture in a point mutagenesis, we first tested its ability to mutate Tyr 66 in the mutant GFPuv to Asp. Because the Tyr 66 residue is key to the formation of the GFPuv chromophore (Figure 2a), the mutation from Tyr to Asp abolished fluorescence. Two PCR fragments were prepared using wild-type GFPuv gene template and two sets of PCR primers (1 and 2 in Additional file 1: Table S2), one of which had a sequence that corresponded to the mutation from the Tyr (TAT) codon to the Asp (GAT) codon. The two fragments were assembled with the NheI–EcoRI-digested pBAD plasmid using MUPAC, and the colonies of the *E. coli* JM109 transformed by the assembled plasmid were exposed to a UV light to observe fluorescence. As expected, none of the colonies exhibited fluorescence, indicating that no detectable wild-type GFPuv gene persisted through MUPAC (Figure 2b vs. 2c). We then introduced a back mutation to the mutant gene to recover GFPuv fluorescence. Two PCR fragments were prepared using mutant GFPuv Y66D template and two sets of PCR primers (1 and 3 in Additional file 1: Table S2), and were assembled with the pBAD plasmid using MUPAC. As a result, 91% of the colonies of the *E. coli* JM109 transformed by the assembled plasmid exhibited fluorescence (Figure 2c vs. 2d). This result shows that a single mutation could be introduced efficiently by MUPAC.

### GFPuv multiple site-directed mutagenesis

Next, we evaluated the performance of MUPAC in multiple mutagenesis. For this experiment, we employed the same back mutation strategy as for GFPuv single site directed mutagenesis to confirm the complete back mutation of mutant GFPuv genes. In addition to the Y66D mutation, four other mutations (V29D, A110D, F165D, and L201D) were chosen because the side chains of these residues were oriented to the inside of the β-barrel structure of GFPuv, and therefore only the one of the mutations could disturb the fluorescent property of the protein (data not shown). For three-point mutagenesis, four PCR fragments were prepared using the V29D-Y66D-A110D mutant (3D-mutant GFPuv) gene template and four sets of PCR primers (4–7 in Additional file 1: Table S2). Alternatively, for five-point mutagenesis, six PCR fragments were prepared using the V29D-Y66D-A110D-F165D-L201D mutant (5D-mutant GFPuv) gene template and six sets of PCR primers (4–6 and 8–10 in Additional file 1: Table S2). These dsDNA fragments were assembled in the NheI–EcoRI-digested pBAD plasmid using MUPAC, and fluorescent and non-fluorescent colonies were counted to calculate the efficiency of complete multiple mutagenesis. Contrary to our expectations, the ratios of the fluorescent and non-fluorescent colonies in three- or five- point mutagenesis (Figure 2e and f) were similar to that in one point mutagenesis (Figure 2d). Conversely, when MISO method was used for five-point mutagenesis by assembling the same plasmid and PCR fragments, only 49% of colonies exhibited fluorescence (Additional file 1: Figure S1). Moreover, the numbers of colonies were similar between the one- (3,240 colonies), three- (3,330 colonies), and five- (3,400 colonies) point mutagenesis by MUPAC. These results clearly demonstrate the high fidelity and efficiency of MUPAC for multiple site-directed mutagenesis.

### Multiple site-directed mutagenesis of M-MLV RT gene

**Figure 3:**
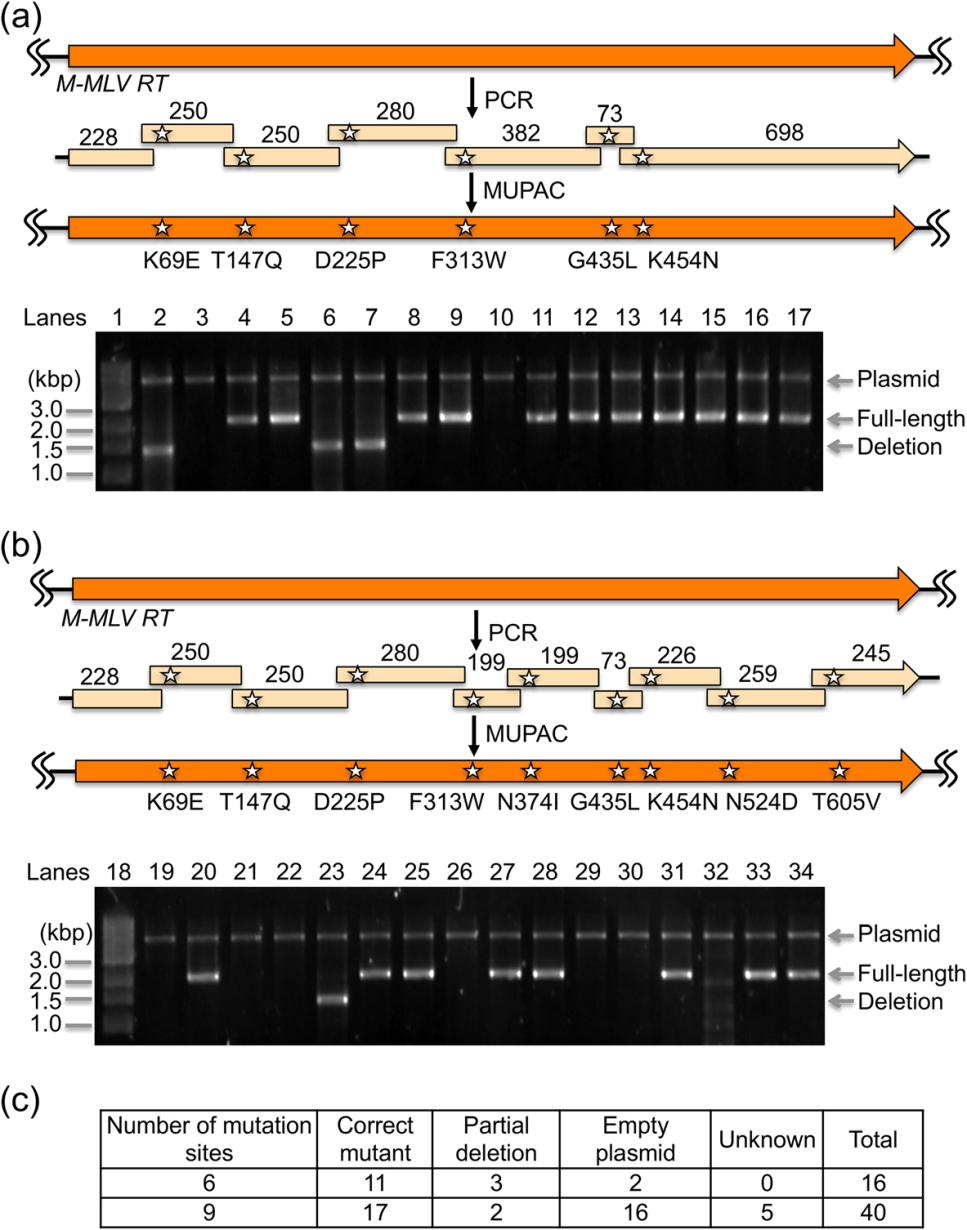
**Multiple site-directed mutagenesis of a M-MLV RT gene.** Schematic presentation of six- **(a)** or nine-point **(b)** simultaneous mutation in M-MLV RT gene is shown. DNA fragments amplified by PCR were assembled with the NdeI–XhoI-digested pET16b vector using MUPAC. Length in base pair is shown over each DNA fragment. Plasmids were extracted from each six- and nine-point site-directed mutagenesis experiment. After digestion with NdeI and XhoI, the digested plasmids were analyzed using 1.2% agarose gel electrophoresis (lanes 2–17, the six-site experiments; lanes 19–34, the nine-site experiment). Lane 1 and 18 contain 1000 bp DNA ladder marker. CFU in each experiment are listed in Additional file 1: Table S3. **(c)** Summary of 6- or 9-points mutation. Mutants were analyzed using electrophoresis and DNA sequencing.

To evaluate the suitability of MUPAC for multiple site-directed mutagenesis, we also attempted to introduce six-point mutations (K69E, T147Q, D225P, F313W, G435L, and K454N) in M-MLV RT gene in the pET16b plasmid. Seven PCR fragments were prepared using the M-MLV RT gene template and seven sets of PCR primers (11–17 in Additional file 1: Table S2; Figure 3a), assembled with NdeI–XhoI-digested pET16b plasmid using MUPAC, and transformed into *E. coli* JM109. Gel electrophoresis of NdeI–XhoI-digested plasmids extracted from the resulting colonies showed that 11 of 16 plasmids possessed full-length M-MLV RT gene (Figure 3a). The DNA sequence analysis of the full-length plasmid also showed that the all the genes sequenced had all six mutations without undesired mutations.

Next, we increased the number of mutation sites from six to nine (K69E, T147Q, D225P, F313W, N374I, G435L, K454N, N524D, and T605V). Ten PCR fragments were prepared using the M-MLV RT gene template and ten sets of PCR primers (11–14, 16, and 18–22 in Additional file 1: Table S2; Figure 3b) were assembled with the NdeI–XhoI-digested pET16b plasmid using MUPAC, and transformed into *E. coli* JM109. Gel electrophoresis of the NdeI–XhoI-digested plasmids showed that 17 of 40 plasmids possessed full-length M-MLV RT gene (Figure 3b, Additional file 1: Figure S2). DNA sequence analysis of the plasmids also showed that all full-length genes had all nine mutations without undesired mutation.

Although the proportion of plasmids possessing the full-length mutant gene decreased as the number of mutation sites increased, MUPAC still produced the desired gene with nine-point mutations in approximately 40% of plasmids (Figure 3c). These results clearly demonstrate the suitability of MUPAC for multiple site-directed mutagenesis.

### Multiple site-directed saturation mutagenesis on GFPuv gene

**Figure 4:**
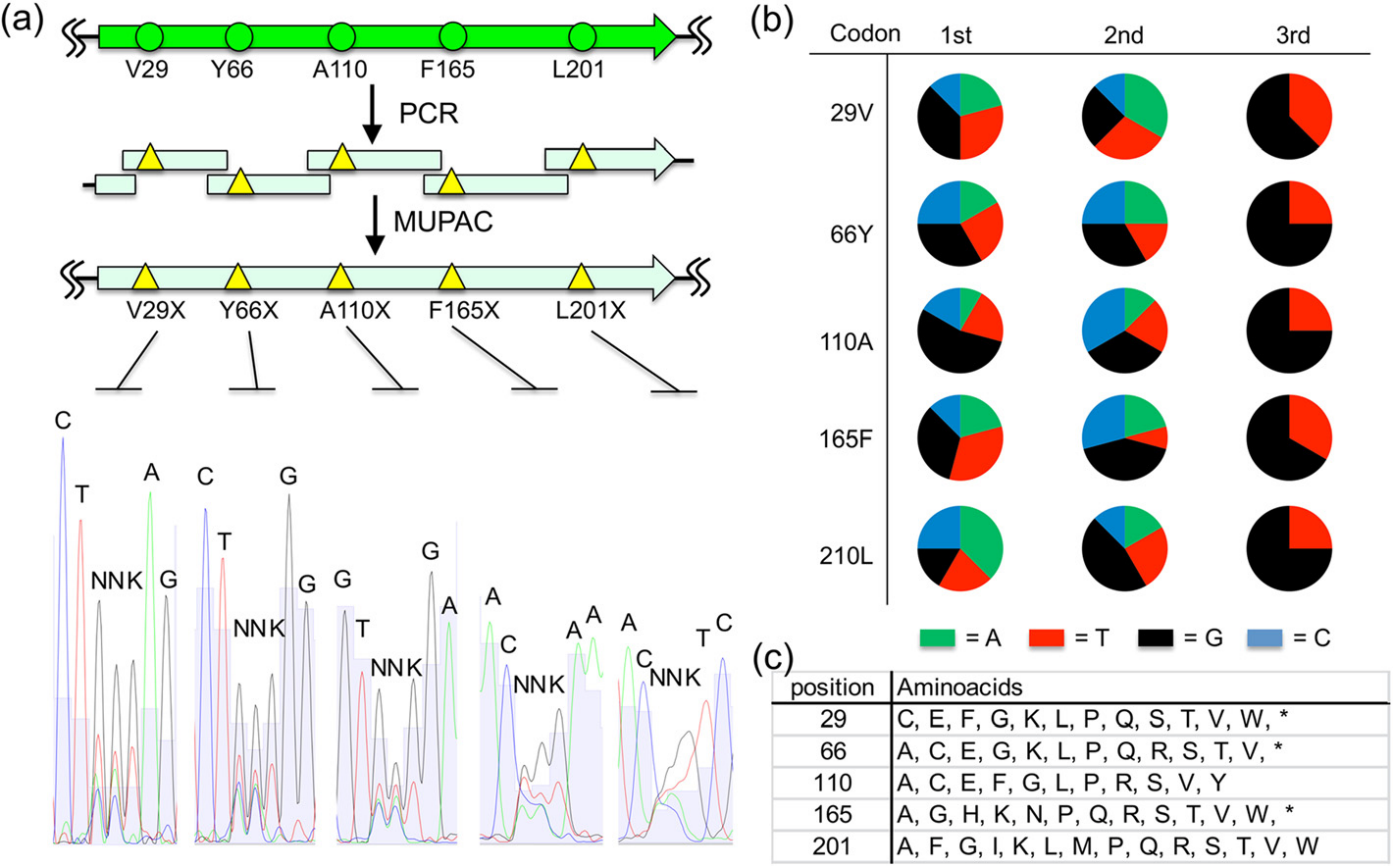
**Site-directed saturation mutagenesis using MUPAC. (a)** DNA sequence chromatogram obtained by sequencing a plasmid mixture from >10^4^ clones. NNK codons, where K represents G or T were simultaneously introduced to the GFPuv gene at the five specific sites by using MUPAC. CFU for the experiment is indicated in Additional file 1: Table S3. **(b)** Distribution of nucleotide bases in the five randomized codons. Twenty-four plasmids obtained from individual clones in the saturation mutagenesis experiment were sequenced. The distribution of nucleotides is shown in pie diagrams for each of the randomized base. The nucleotide base A is depicted in green, T in red, G in black, and C in blue. **(c)** Amino acids appeared at the five sites. Twenty-four plasmids obtained from individual clones in the saturation mutagenesis experiment were sequenced. Translated amino acids at the five sites are shown in one-letter style, and stop codon is denoted by asterisk.

A number of studies have shown that the site-directed saturation mutagenesis is useful for altering or improving a protein function [1-13]. Therefore, we tested the performance of MUPAC in five-point site-directed saturation mutagenesis (V29X, Y66X, A110X, F165X, and L201X; X represents one of the 20 proteinogenic amino acids) of GFPuv gene. Six PCR fragments, each of them bearing NNK codon at the desired site, were prepared using GFPuv gene and six sets of PCR primers (4, 23–27 in Additional file 1: Table S2; Figure 4a). The resulting dsDNA fragments were assembled in an NheI–EcoRI-digested pBAD plasmid using MUPAC, and transformed into *E. coli* DH10B by electroporation. Sequence analysis of the plasmid mixture extracted from >10^4^ of the resulting colonies showed that the extracted genes contained random NNK codons at the desired sites (Figure 4a and Additional file 1: Figure S3). We also independently sequenced 24 plasmids extracted from 24 randomly chosen colonies with GFPuv gene as an insert (Additional file 1: Table S4). The sequence data showed that all plasmids had mutations only at the desired sites. At each mutation site, the codons exhibited A, T, G, or C as the first and second bases, and T or G as the third base (Figure 4b), and an average of 13 amino acid variations (including stop codon) was found at each site of the NNK codon (Figure 4c). These results demonstrate the suitability of MUPAC for the preparation of a gene pool with multiple site-directed saturated mutations.

## Discussion

We developed a DNA assembly method, termed MUPAC, capable of assembling multiple dsDNAs with short homologous sequences (Figure 1). Unlike the ISO method, MUPAC uses DNA modification enzymes with an optimum reaction temperature of 37°C. This feature allows the use of short (e.g., 16 bp) homologous assembly sequences, and confers high assembly efficiency.

We first used MUPAC for the back mutation of fluorescence-deficient mutant GFPuv with one-, three-, or five-site mutations (in which the original residue was replaced by Asp). In all cases, approximately 90% of the resulting colonies exhibited GFPuv fluorescence, which demonstrates a high fidelity of MUPAC for multiple site-directed mutagenesis (Figure 2d, e, f). This result also demonstrated the high efficiency of MUPAC because the similar numbers of colonies were obtained for one-, three-, or five-point site-directed mutagenesis of GFPuv.

To explain why 10% of colonies were non-fluorescent, we extracted plasmids from the four non-fluorescent colonies formed in the five-point site-directed mutagenesis experiment, and we analyzed them using electrophoresis and DNA sequencing (Additional file 1: Figure S4). One contained a single nucleotide deletion in the primer region, indicating that impurity of the synthesized primer caused the production of an undesired product. The other three plasmids showed higher mobility than the pBAD-NXE plasmid on electrophoresis analysis, suggesting that the over-degradation of the plasmid with T5 exonuclease caused the production of the undesired small plasmid.

We also found that the concentration of T5 exonuclease, Klenow fragment exo - and T4 DNA ligase influenced the efficiency of DNA assembly (Additional file 1: Figures S5 and S6). For efficient DNA assembly, at least 0.1 U/μL of T5 exonuclease was required. Assembly efficiency was influenced to a lesser degree by the concentration of Klenow fragment exo^–^, although the excess of the Klenow fragment exo^–^ caused a significant reduction in the number of colonies carrying the desired plasmid. Higher (2×) concentration of T4 DNA ligase did not alter the assembly efficiency (520 colonies and 530 colonies), but slight reduction in the number of colonies was observed when the concentration was reduced to half the concentration (440 colonies; Additional file 1: Figure S6).

To demonstrate the suitability of MUPAC for multiple site-directed mutagenesis, we performed six- and nine-point site-directed mutagenesis of M-MLV RT gene. Analysis by electrophoresis showed that the 11 of 16 (for six-point mutagenesis) and 17 of 40 (for nine-point mutagenesis) plasmids possessed full-length M-MLV RT gene as an insert (Figure 3). Sequence analysis of the plasmids containing the full-length gene showed that all the plasmids analyzed had all, six or nine, desired mutations.

In contrast with GFPuv mutagenesis, pET16b plasmid was found in 2 of 16 colonies (for six-point mutagenesis) and 19 of 40 colonies (for nine-point mutagenesis) in M-MLV RT mutagenesis. Similar results were obtained between experiments using different lots of restriction enzyme-digested pET16b (Figure 3b vs. Additional file 1: Figure S2). We sequenced plasmids with truncated M-MLV RT gene; all possessed a deleted sequence in the same position (Additional file 1: Figure S7). This deletion was generated by an undesired assembly with a 7-bp homologous region. Because M-MLV RT gene has 128 of 7-bp, 30 of 8-bp, 15 of 9-bp, and 1 of 10-bp homologous regions (Additional file 1: Table S5), it is surprising that only one deleted sequence resulted from an undesired assembly.

The increased proportion of undesired plasmids with nine-point mutagenesis compared with six-point mutagenesis suggests that the number of DNA fragments requiring assembly is a limitation of MUPAC. Similarly, when MUPAC was used to assemble eight DNA fragments of M-MLV RT gene, one of which was 49-bp in length, the proportion of the plasmids carrying the desired sequence significantly decreased (data not shown). Because T5 exonuclease degrades the DNA in the 5′–3′ direction at the each end, short DNA fragments could simply be decomposed before assembly. The shortest DNA fragment we used in this experiment was 73 bp; therefore, the lower limit of the DNA length could be 50–70 bp. Thus, when the distance between two mutation sites is under 50 bp, we recommend using a pair of overlapping forward and reverse primers, both of which carry each mutation (Additional file 1: Figure S8a). This is also applicable to mutations whose interval is as short as 16 bp; when the distance between two mutation sites is less than 16 bp, a longer primer with two mutations should be used (Additional file 1: Figure S8b).

We also applied MUPAC in multiple site-directed saturation mutagenesis. Five sites of GFPuv gene were chosen to introduce an NNK codon, and the plasmids extracted from the mutant gene pool were sequenced. All genes contained mutations at the desired sites, and no undesired mutations existed elsewhere (Figure 4a, Additional file 1: Figure S3). N (A, T, G, or C) at the first and second bases and K (T or G) at the third base of the NNK codons were evident in all codons at the five desired sites, although the G appeared more frequently (Figure 4b). This bias toward G could be simply caused by the quality of the random oligonucleotides purchased from Operon Biotechnologies because a similar bias was observed in different oligonucleotides purchased from the same company (data not shown).

## Conclusions

We developed the MUPAC system that could efficiently assemble multiple DNAs with a short homologous sequence. MUPAC successfully achieved one- to five-point site-directed mutagenesis of the GFPuv, and six- or nine-point site-directed mutagenesis of the M-MLV RT gene. Furthermore, we demonstrated the suitability of MUPAC for five-point site-directed saturation mutagenesis of the GFPuv gene. We have also used this method for the simple cloning of a gene, and for construction of a plasmid with tandemly arranged genes (unpublished result). Because MUPAC can flexibly manipulate DNA in a simple and efficient manner, this method could greatly contribute toward research in many fields including molecular and synthetic biology.

## Methods

### Plasmids

The plasmid pBAD-NXE was constructed by the insertion of double-stranded DNA (dsDNA) between NheI and EcoRI sites of pBAD-GFPuv (Bio-Rad, Hercules, CA) [23]. The DNA sequence between the NheI and EcoRI sites of pBAD-NXE was 5′-ATTAA CTCGA GCTTA T -3′. Mutant Moloney Murine Leukemia Virus reverse transcriptase gene (M-MLV RT, Additional file 1: Figure S9) [24,25] was purchased from Operon Biotechnologies (Alameda, CA) and was cloned into pET16b (Novagen, Darmstadt, Germany) between NdeI and XhoI sites to construct pET16b-MMLVRT.

### Site-directed multiple mutagenesis

The template plasmids (pBAD-GFPuv and pET16b-MMLVRT) were digested with EcoRV (New England Biolabs, Ipswich, MA) in the presence of alkaline phosphatase (CIP, New England Biolabs) in order to avoid contamination with the original plasmid. Templates were added to the PCR reaction mixture (20 μL, KOD DNA polymerase; Toyobo, Tokyo, Japan) without purification, and the DNA fragments were amplified by PCR using the pairs of the forward and reverse primers (Additional file 1: Tables S1 and S2). All DNA fragments were purified by using a FastGene Gel/PCR Extraction Kit (Nippon Gene, Toyama, Japan), and eluted with 20 μL of the elution buffer.

The plasmids pBAD-NXE and pET16b were digested with NheI–EcoRI and NdeI–XhoI, respectively, in the presence of CIP. The products were purified by using the FastGene Gel/PCR Extraction Kit.

The MUPAC enzyme stock solution was prepared by mixing 10 μL of T5 exonuclease (10 U/μL; New England Biolabs), 1 μL of Klenow fragment exo^–^ (5 U/μL; New England Biolabs), 2.5 μL of T4 DNA ligase (400 U/μL; New England Biolabs), and 11.5 μL of the storage buffer (50 mM Tris–HCl pH 7.5, 100 mM NaCl, 1 mM DTT, 0.1 mM EDTA, 50% Glycerol, and 0.1% Triton X-100). The enzyme stock solution was stored at –20°C. The MUPAC reagent (2×) was prepared just prior to use by mixing 1 μL of the enzyme stock solution and 20 μL of the reaction buffer (100 mM Tris–HCl pH 7.5, 20 mM MgCl_2_, 20 mM DTT, 2 mM ATP, 0.5 mM dNTPs, and 0.02% BSA).

The MUPAC reaction mixture was prepared by mixing the restriction enzyme-digested plasmid (0.2 μL), PCR-amplified DNA (0.8 μL), and MUPAC reagent (2×; 1 μL). The mixture (2 μL; comprising 5 ng/μL of restriction enzyme-digested plasmid, 3–8 ng/μL DNA fragments, 50 mM Tris–HCl pH 7.5, 10 mM MgCl_2_, 10 mM DTT, 1 mM ATP, 0.25 mM dNTPs, 0.01% BSA, 0.1 U/μL T5 exonuclease, 0.005 U/μL Klenow fragment exo^–^, and 1 U/μL T4 DNA Ligase) was incubated at 37°C for 30 min and used directly for chemical transformation of *E. coli* JM109.

### GFPuv fluorescence measurement

After the transformation, the solution was diluted with LB medium and plated onto an LB agar plate containing 50 μg/mL ampicillin and 0.2% arabinose. After 16 h of incubation at 37°C, GFPuv fluorescence was observed through a Y48 filter (480 nm cutoff filter; HOYA, Tokyo, Japan) under 365 nm UV light.

### Analysis of mutant M-MLV RT genes

After transformation, the solution was diluted with LB medium and plated onto an LB agar plate containing 50 μg/mL ampicillin. Plasmids were extracted from 2 mL of a solution containing JM109 transformants cultured overnight at 37°C. The plasmids were digested with NdeI and XhoI, and analyzed by agarose gel electrophoresis. The DNA sequences of the plasmids were determined using the dideoxyribonucleotide method.

### Site-directed multiple saturation mutagenesis

The MUPAC reaction was performed using the same procedure as mentioned above. Subsequently, the DNA was purified by ethanol precipitation, and used to transform *E. coli* DH10B by electroporation. Plasmids were extracted from 2 mL of a solution containing DH10B transformants cultured overnight, and the DNA sequences were determined by the dideoxyribonucleotide method. Alternatively, a DNA sequence chromatogram was obtained from the mixture of the plasmids extracted from all colonies (> 10^4^) on an LB agar plate.

## Competing interests

The authors declare that they have no competing interests.

## Authors’ contributions

NT and HM participated in the design of the study and performed the experiments. SN and TK participated in the optimization of the reaction conditions of MUPAC. All authors read and approved the final manuscript.

## Supplementary Material

Additional file 1: Figure S1 Back mutation of 5D-mutant GFPuv gene by using MISO method. **Figure S2.** Nine-point mutagenesis of M-MLV RT gene. **Figure S3.** DNA sequence chromatogram from the analysis of saturation mutant pool. **Figure S4.** Electrophoresis and sequence analysis of plasmids extracted from non-fluorescent colonies in the experiment of the five-point mutagenesis of the 5D-GFPuv gene. **Figure S5.** Effect of T5 Exonuclease and Klenow Fragment concentrations on the efficiency of MUPAC. **Figure S6.** Effect of T4 DNA ligase concentration on the efficiency of MUPAC. **Figure S7.** Undesired assembly of DNA fragments that found in the nine-point mutagenesis experiment of the M-MLV RT gene. **Figure S8.** Two ways to introduce the mutations, whose distance were less than 50 bp. **Figure S9.** The sequence of the mutant M-MLV reverse transcriptase gene. **Table S1.** Oligonucleotide DNAs used in this study. **Table S2.** Primer sets of oligonucleotide DNAs used in each experiment and the length of the amplified DNA fragment. **Table S3.** Calculated CFU (colony forming unit) in each experiment. **Table S4.** List of codons at the five randomized sites of GFPuv. **Table S5.** Number of intragene homologous sequences. Click here for file
