# Supplementary material for: Patch cloning method for multiple site-directed and saturation mutagenesis
Source: BMC Biotechnol. 2013 Oct 29;13:91. doi: 10.1186/1472-6750-13-91 (PMC3829206; doi:10.1186/1472-6750-13-91)
Supplement: Additional file 1: Figure S1 — Back mutation of 5D-mutant GFPuv gene by using MISO method. Figure S2. Nine-point mutagenesis of M-MLV RT gene. Figure S3. DNA sequence chromatogram from the analysis of saturation mutant pool. Figure S4. Electrophoresis and sequence analysis of plasmids extracted from non-fluorescent colonies in the experiment of the five-point mutagenesis of the 5D-GFPuv gene. Figure S5. Effect of T5 Exonuclease and Klenow Fragment concentrations on the efficiency of MUPAC. Figure S6. Effect of T4 DNA ligase concentration on the efficiency of MUPAC. Figure S7. Undesired assembly of DNA fragments that found in the nine-point mutagenesis experiment of the M-MLV RT gene. Figure S8. Two ways to introduce the mutations, whose distance were less than 50 bp. Figure S9. The sequence of the mutant M-MLV reverse transcriptase gene. Table S1. Oligonucleotide DNAs used in this study. Table S2. Primer sets of oligonucleotide DNAs used in each experiment and the length of the amplified DNA fragment. Table S3. Calculated CFU (colony forming unit) in each experiment. Table S4. List of codons at the five randomized sites of GFPuv. Table S5. Number of intragene homologous sequences. [file 1472-6750-13-91-S1.docx]

**Additional file 1**

**Patch cloning method for multiple site-directed mutagenesis and saturation mutagenesis**

Naohiro Taniguchi, Sayumi Nakayama, Takashi Kawakami, and Hiroshi Murakami*

Department of Life Sciences, Graduate School of Arts and Sciences, The University of Tokyo, 3-8-1 Komaba, Meguro-ku, Tokyo, 153-8902, Japan

* To whom correspondence should be addressed: +81-3-5465-8850, murah@bio.c.u-tokyo.ac.jp

**
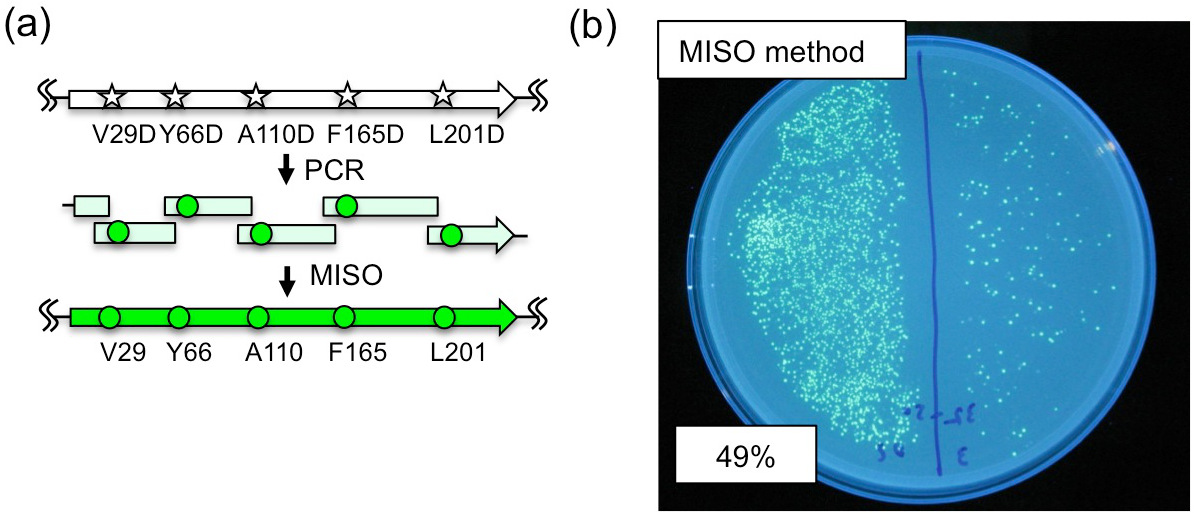
**

**Figure S1.** Back mutation of 5D-mutant GFPuv gene by using MISO method. (a) Schematic illustration of back mutation of 5D-mutant GFPuv gene by using MISO method. (b) PCR-amplified six DNA fragments and pBAD vector digested with NheI and EcoRI were assembled by using MISO method, and the resulting was transformed into *E. coli* JM109. A tenth aliquot of transformants were spread on the right half of LB agar plate and the rest on the left half. Percentage frequency of fluorescent colonies is indicated on the lower left.

**
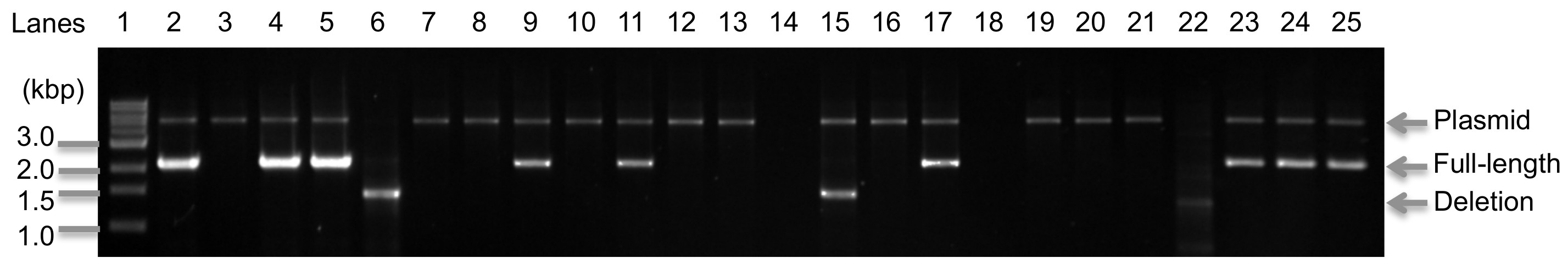
**

**Figure S2.** Nine-point mutagenesis of M-MLV RT gene. Nine-point simultaneous mutation on M-MLV RT is performed and 24 resultant plasmids were analyzed as shown in Fig. 3b. 1000-bp DNA ladder marker is shown in lane 1.

**
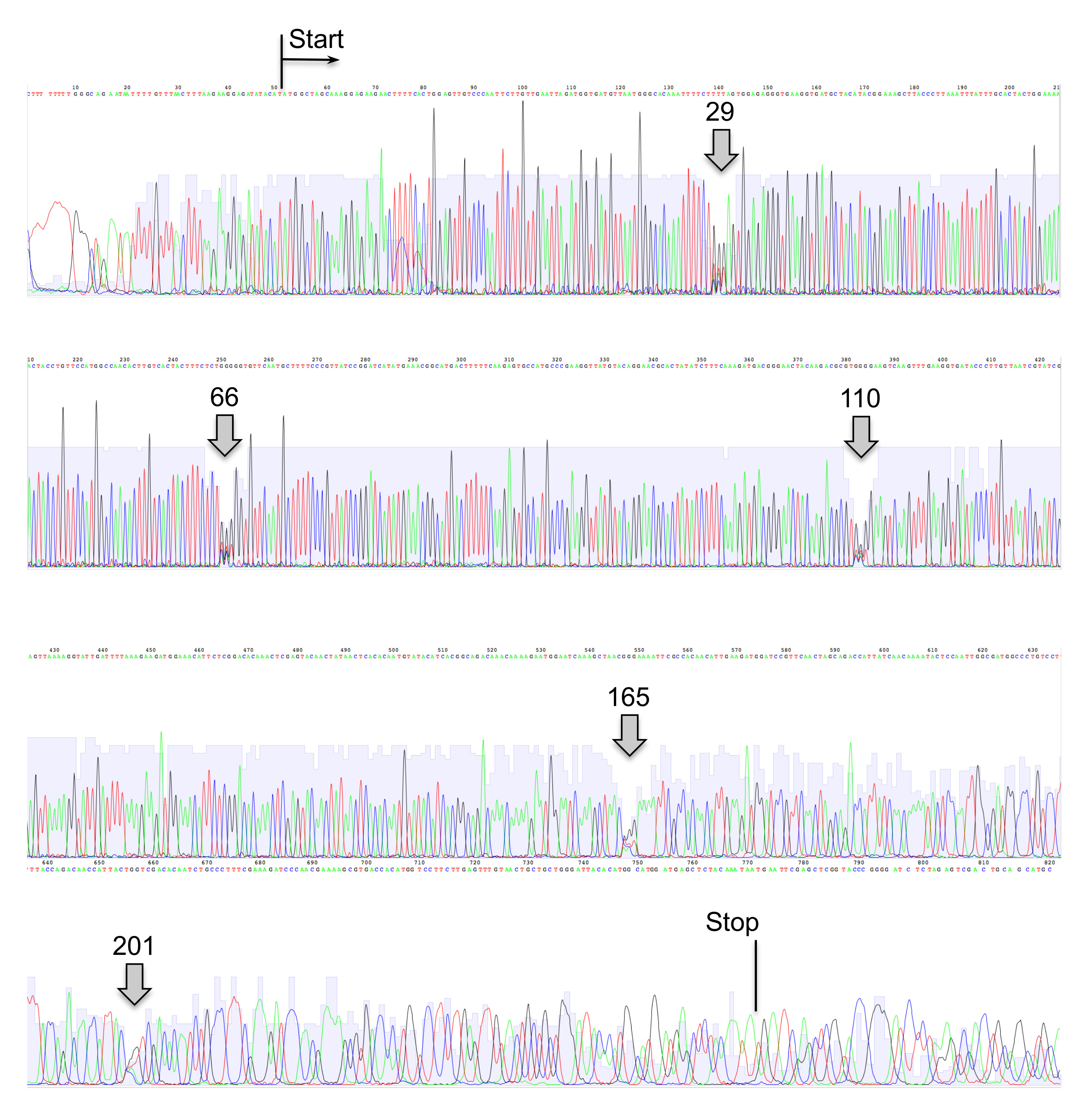
**

**Figure S3.** DNA sequence chromatogram from the analysis of saturation mutant pool. Site-directed saturation mutagenesis and DNA sequencing were performed as described in Fig. 4a. Whole chromatogram is shown in the figure.

**
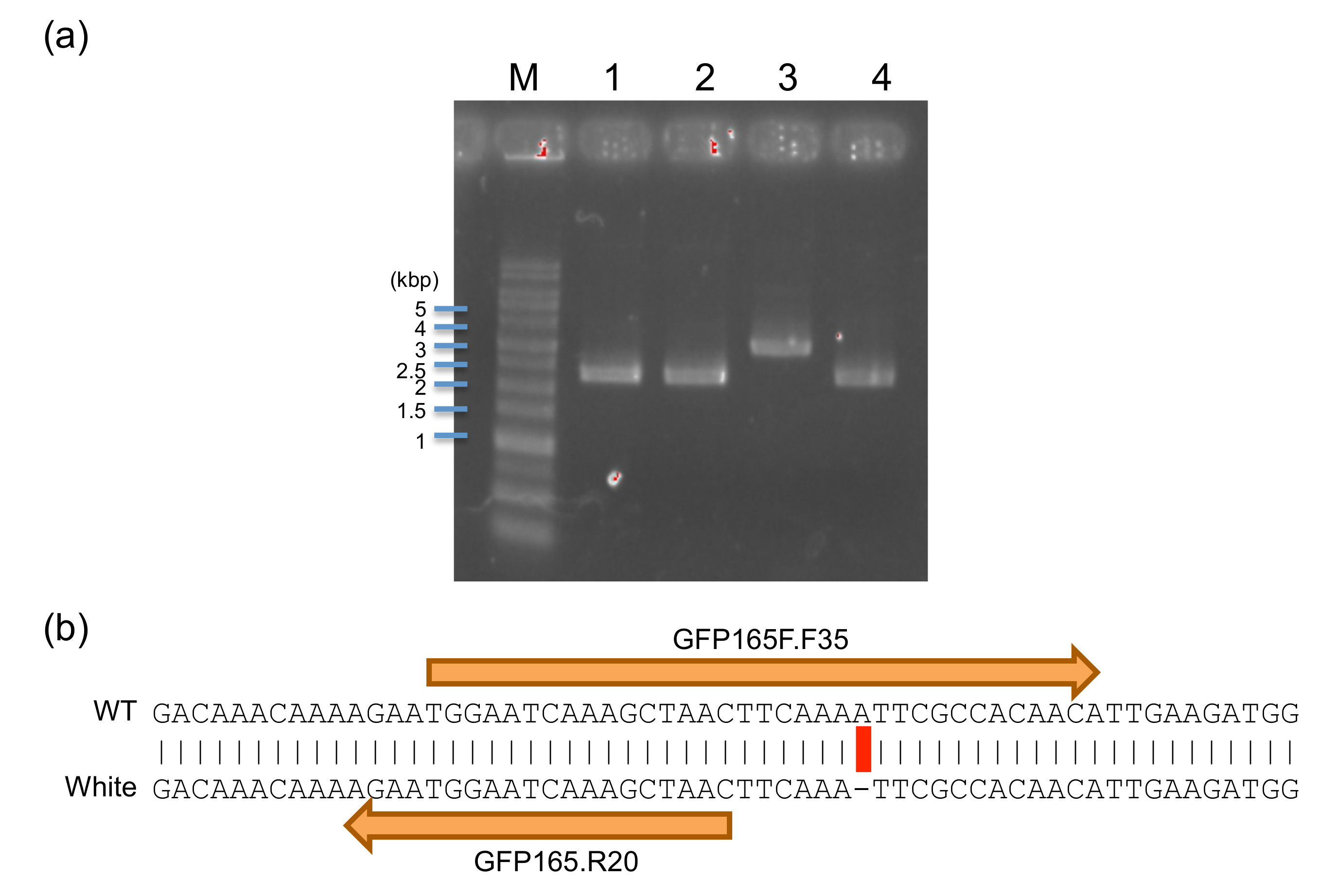
**

**Figure S4.** Electrophoresis and sequence analysis of plasmids extracted from non-fluorescent colonies in the experiment of the five-point mutagenesis of the 5D-GFPuv gene. (a) Agarose gel electrophoresis analysis of the plasmids extracted from four non-fluorescent colonies. (b) DNA sequencing result of plasmid DNA from the lane 3 in (a). Annealing sites of MUPAC primers GFP165F.F35 and GFP165.R20 are shown.


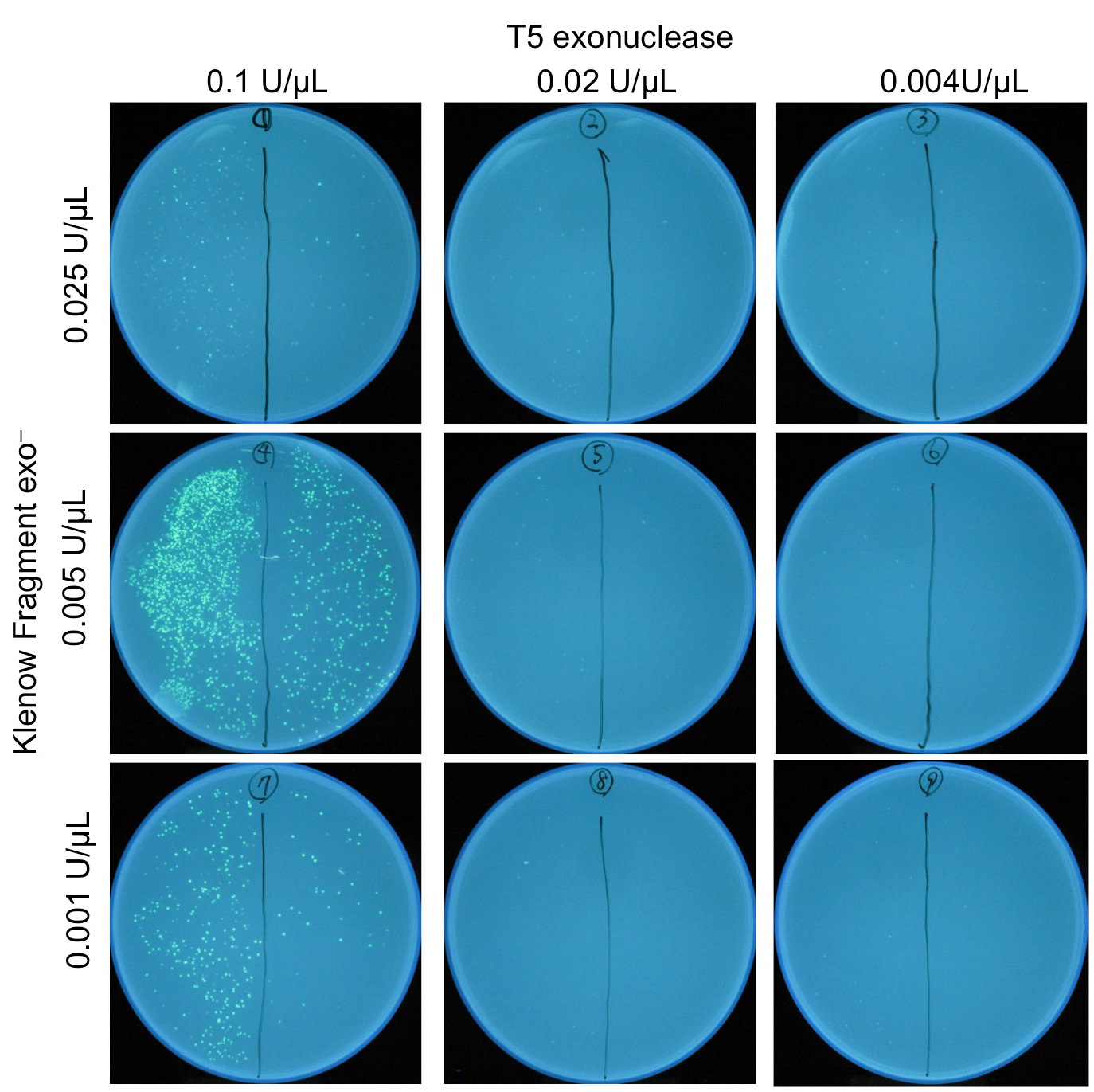


**Figure S5.** Effect of T5 Exonuclease and Klenow Fragment concentrations on the efficiency of MUPAC. Back mutation experiments of the 5D-mutant GFPuv gene using MUPAC were performed with various concentrations of Klenow fragment and T5 exonuclease as indicated. A tenth aliquot of transformant *E. coli* JM109 were spread on the right half of LB agar plate and the rest on the left half.

**
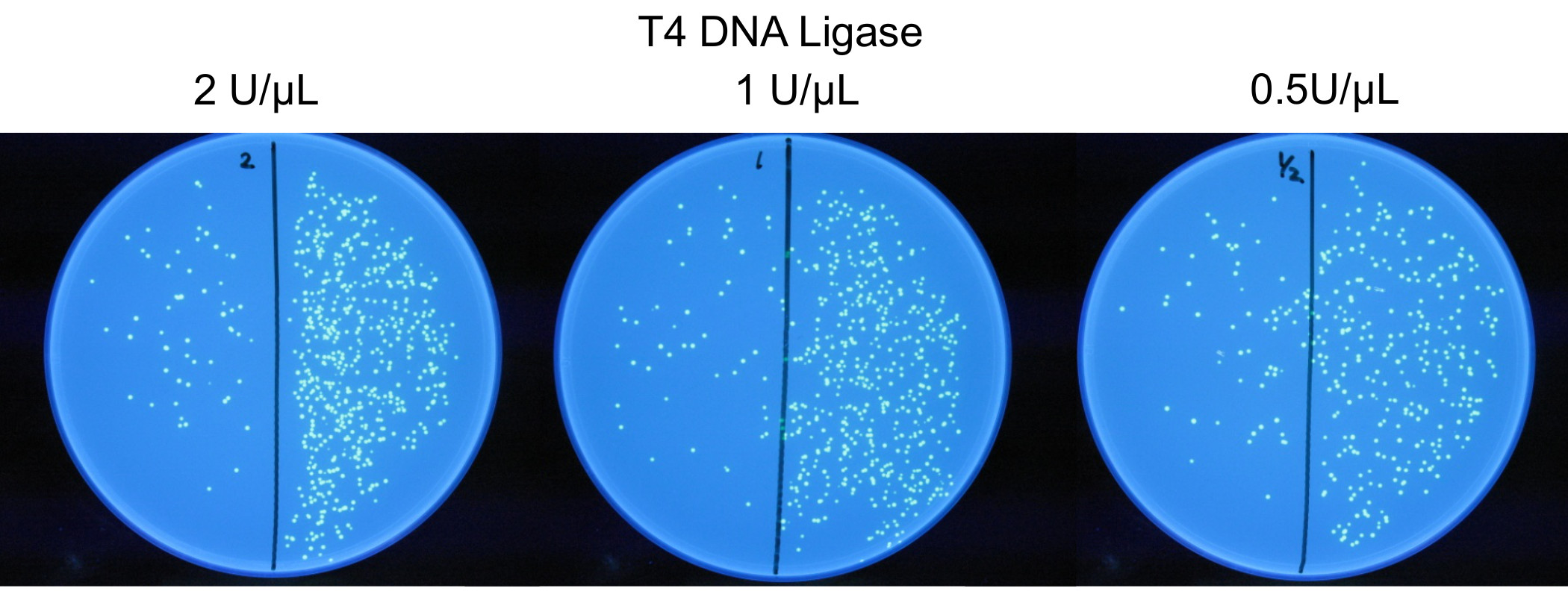
**

**Figure S6.** Effect of T4 DNA ligase concentration on the efficiency of MUPAC. Back mutation experiments of the 5D-mutant GFPuv gene using MUPAC were performed with 2 U/μL, 1 U/μL, or 0.5 U/μL of T4 DNA ligase. A tenth aliquot of transformant *E. coli* JM109 was spread on the left half of LB agar plate and the rest on the right half.


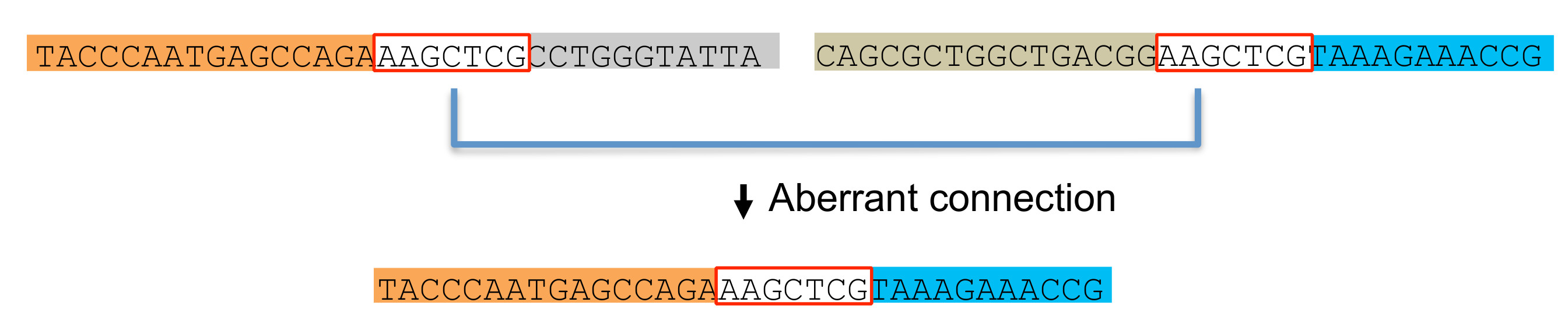


**Figure S7.** Undesired assembly of DNA fragments that found in the nine-point mutagenesis experiment of the M-MLV RT gene. DNA sequences of M-MLV RT 193–226 (left) and 832–865 (right) are shown. 7-base-pair homologous regions are highlighted with red rectangles. Connection of DNA fragments in the 7-bp homologous regions resulted in partial deletant.


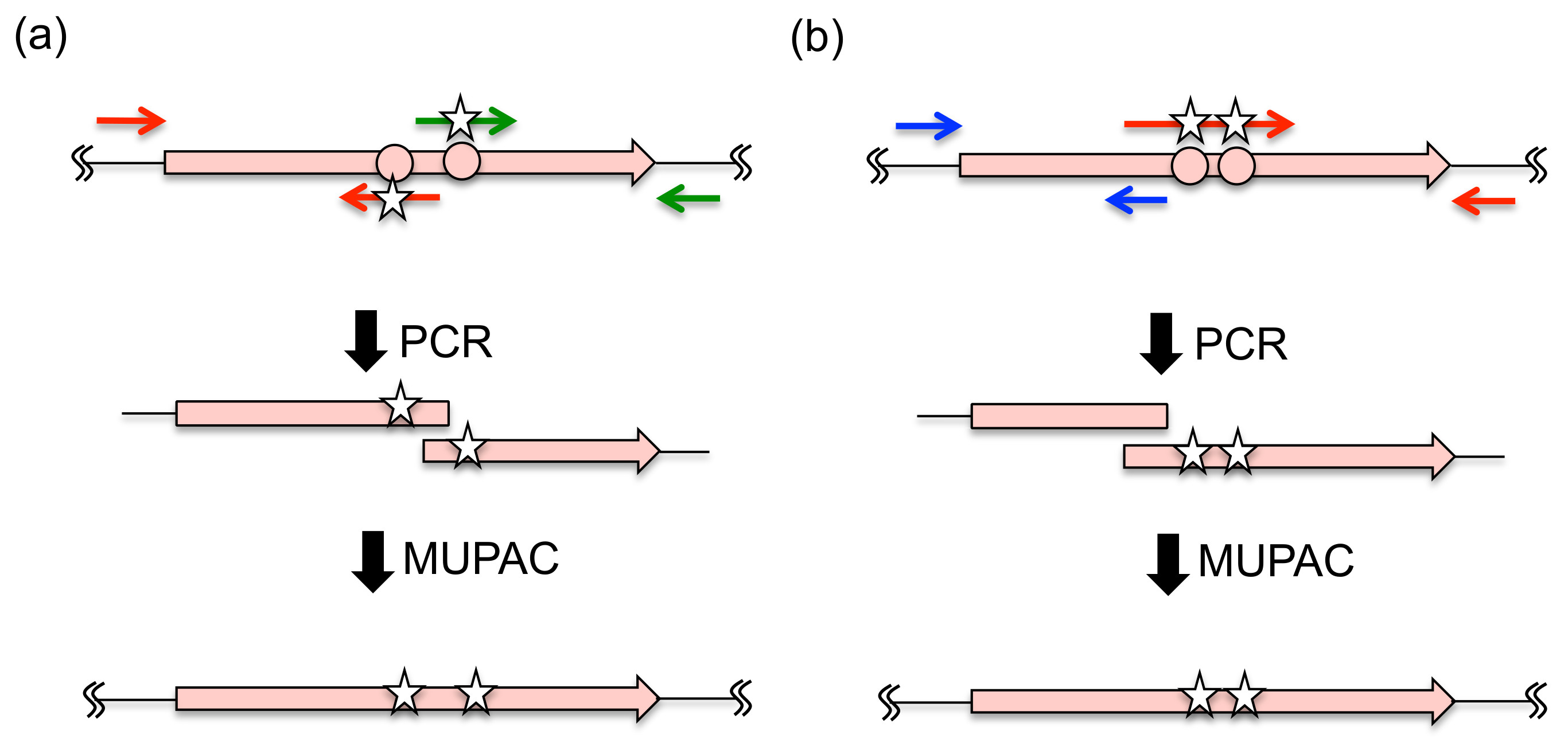


**Figure S8.** Two ways to introduce the mutations, whose distance were less than 50 bp. (a) Introducing two mutations with overlapping forward and reverse primers. (b) Introducing two mutations with a single primer.

CATATGACTCTGAACATTGAGGATGAACACCGTTTACACGAAACGAGCAAAGAACCGGATGTATCGCTGGGCTCGACGTGGCTGTCTGACTTTCCTCAGGCGTGGGCAGAAACCGGCGGTATGGGTCTGGCGGTACGTCAAGCCCCGCTGATCATCCCACTTAAAGCTACATCGACCCCGGTCAGCATCAAACAGTACCCAATGAGCCAGAAAGCTCGCCTGGGTATTAAACCGCATATTCAGCGCCTGCTGGATCAGGGCATCCTGGTCCCGTGTCAGAGTCCGTGGAATACCCCGTTACTCCCAGTGAAGAAACCGGGAACGAATGACTATCGCCCGGTTCAGGACCTGCGTGAGGTGAACAAACGCGTTGAGGACATTCACCCAACCGTCCCTAATCCGTATAACTTGCTGTCAGGTTTGCCGCCGAGCCATCAGTGGTACACCGTTTTGGACCTGAAAGATGCCTTCTTTTGCCTTCGGCTGCATCCGACATCTCAACCGCTCTTTGCGTTTGAATGGCGTGACCCGGAAATGGGAATCTCTGGTCAGCTGACGTGGACTCGCCTGCCGCAAGGCTTTAAGAACTCCCCAACTCTGTTCGATGAAGCGCTGCATCGGGACTTGGCCGATTTCCGCATTCAACACCCAGATCTTATTCTGCTGCAGTACGTGGACGACCTCTTGCTGGCAGCCACTAGCGAACTGGATTGCCAACAGGGAACCCGCGCTCTGTTGCAGACACTGGGCAATCTGGGCTATCGCGCATCGGCGAAGAAAGCTCAGATTTGCCAGAAACAGGTGAAATACTTAGGTTACCTGCTTAAAGAAGGTCAGCGCTGGCTGACGGAAGCTCGTAAAGAAACCGTGATGGGCCAACCGACGCCTAAGACGCCTCGTCAGTTGCGGCGCTTTCTCGGGACTGCGGGGTTCTGTCGCCTGTTCATCCCGGGATTTGCGGAGATGGCGGCACCCCTGTATCCGCTGACTAAGACGGGTACCTTATTCAACTGGGGTCCCGATCAGCAGAAAGCCTATCAGGAGATTAAACAAGCTCTGTTAACAGCGCCGGCCTTGGGCTTACCCGATCTGACCAAACCGTTCGAGTTATTCGTTGATGAGAAACAAGGCTATGCCAAAGGCGTACTCACCCAGAAATTGGGTCCGTGGCGTCGTCCAGTCGCGTATCTGTCCAAAAAACTCGACCCCGTTGCGGCAGGGTGGCCGCCTTGTCTGCGGATGGTCGCGGCTATTGCAGTGCTGACCAAGGATGCAGGCAAACTGACGATGGGTCAACCCCTTGTGATTGGCGCACCACACGCTGTAGAAGCACTGGTGAAACAGCCTCCGGATCGTTGGCTGAGTAAAGCCCGCATGACCCATTATCAAGCGCTGCTGCTGGATACCGATCGCGTTCAATTTGGGCCTGTGGTGGCACTCAATCCCGCCACACTGCTGCCATTACCGGAAGAGGGCCTGCAGCACAATTGCCTGGACATTCTGGCCGAAGCGCATGGTACCCGTCCGGATCTGACCGATCAGCCCCTTCCGGATGCGGATCATACCTGGTACACGAACGGTTCATCCCTGCTCCAAGAAGGCCAACGTAAAGCTGGCGCAGCTGTGACGACCGAAACCGAAGTCATCTGGGCAAAAGCGTTGCCTGCTGGCACCAGTGCCCAGCGCGCAGAACTTATTGCGTTGACGCAGGCCCTTAAAATGGCCGAGGGTAAGAAACTGAACGTTTACACTGATAGTCGCTATGCCTTTGCCACGGCACACATTCACGGAGAAATCTATCGCCGTCGTGGTCTGCTTACCTCAGAGGGGAAAGAAATCAAGAACAAGGACGAAATCTTAGCGCTCTTGAAGGCGCTCTTTCTGCCAAAACGCTTAAGCATTATCCATTGTCCGGGGCATCAGAAAGGCCATTCCGCCGAAGCGCGCGGCAATCGCATGGCGGACCAAGCCGCCCGTAAAGCGGCGATTACCGAAACCCCTGATACTAGCACATTATTATAACTCGAG

**Figure S9.** The sequence of the mutant M-MLV reverse transcriptase gene. NdeI and XhoI sites are underlined. **Table S1.** Oligonucleotide DNAs used in this study. The sequences are written from left to right in the 5′ to 3′ direction. Mutation site is shown in Italic. Oligonucleotide DNAs were purchased from Greiner Bio-One or Operon.

| Name | Sequences (5′ to 3′ direction) |
| --- | --- |
| pBADGFPmut2.F20 | AGAAGGAGATATACATATGG |
| pBADGFPmut4.R18 | ATCCCCGGGTACCGAGCT |
| GFP66D.F35 | TGTCACTACTTTCTCT *GAT* GGTGTTCAATGCTTTT |
| GFP29V.F35 | TGGGCACAAATTTTCT *GTC* AGTGGAGAGGGTGAAG |
| GFP66Y.F35 | TGTCACTACTTTCTCT *TAT* GGTGTTCAATGCTTTT |
| GFP110A.F35 | GAACTACAAGACGCGT *GCT* GAAGTCAAGTTTGAAG |
| GFP165F.F35 | TGGAATCAAAGCTAAC *TTC* AAAATTCGCCACAACA |
| GFP201L.F35 | ACCAGACAACCATTAC *CTG* TCGACACAATCTGCCC |
| GFP29.R20 | AGAAAATTTGTGCCCATTAA |
| GFP66.R20 | AGAGAAAGTAGTGACAAGTG |
| GFP110.R20 | ACGCGTCTTGTAGTTCCCGT |
| GFP165.R20 | GTTAGCTTTGATTCCATTCT |
| GFP201.R20 | GTAATGGTTGTCTGGTAAAA |
| pET16bMMLVmut.F20 | GGCCATATCGAAGGTCGTCA |
| pET16bMMLVmut.R20 | CTTTGTTAGCAGCCGGATCC |
| MMLV69E.F35 | GTACCCAATGAGCCAG *GAA* GCTCGCCTGGGTATTA |
| MMLV147Q.F35 | GAGCCATCAGTGGTAC *CAG* GTTTTGGACCTGAAAG |
| MMLV225P.F35 | GCTGCAGTACGTGGAC *CCG* CTCTTGCTGGCAGCCA |
| MMLV313W.F35 | GGGGTTCTGTCGCCTG *TGG* ATCCCGGGATTTGCGG |
| MMLV374I.F35 | ATTCGTTGATGAGAAA *ATC* GGCTATGCCAAAGGCG |
| MMLV435L.F35 | TCAACCCCTTGTGATT *CTG* GCACCACACGCTGTAG |
| MMLV454N.F35 | GGATCGTTGGCTGAGT *AAC* GCCCGCATGACCCATT |
| MMLV524D.F35 | TCATACCTGGTACACG *GAC* GGTTCATCCCTGCTCC |
| MMLV605V.F35 | CCGTCGTGGTCTGCTT *GTT* TCAGAGGGGAAAGAAA |
| MMLV69.R20 | CTGGCTCATTGGGTACTGTT |
| MMLV147.R20 | GTACCACTGATGGCTCGGCG |
| MMLV225.R20 | GTCCACGTACTGCAGCAGAA |
| MMLV313.R20 | CAGGCGACAGAACCCCGCAG |
| MMLV374.R20 | TTTCTCATCAACGAATAACT |
| MMLV435.R20 | AATCACAAGGGGTTGACCCA |
| MMLV454.R20 | ACTCAGCCAACGATCCGGAG |
| MMLV524.R20 | CGTGTACCAGGTATGATCCG |
| MMLV605.R20 | AAGCAGACCACGACGGCGAT |
| GFP29X.F35 | TGGGCACAAA TTTTCT *NNK* AGTGGAGAGGG TGAAG |
| GFP66X.F35 | TGTCACTACT TTCTCT *NNK* GGTGTTCAATG CTTTT |
| GFP110X.F35 | GAACTACAAG ACGCGT *NNK* GAAGTCAAGTT TGAAG |
| GFP165X.F35 | TGGAATCAAA GCTAAC *NNK* AAAATTCGCCA CAACA |
| GFP201X.F35 | ACCAGACAAC CATTAC *NNK* TCGACACAATC TGCCC |

**Table S2.** Primer sets of oligonucleotide DNAs used in each experiment and the length of the amplified DNA fragment.

| Primer set number | Forward Primer | Reverse Primer | Fragment Size (bp) |
| --- | --- | --- | --- |
| 1 | pBADGFPmut2.F20 | GFP66.R20 | 214 |
| 2 | GFP66D.F35 | pBADGFPmut4.R18 | 564 |
| 3 | GFP66Y.F35 | pBADGFPmut4.R18 | 564 |
| 4 | pBADGFPmut2.F20 | GFP29.R20 | 103 |
| 5 | GFP29V.F35 | GFP66.R20 | 127 |
| 6 | GFP66Y.F35 | GFP110.R20 | 148 |
| 7 | GFP110A.F35 | pBADGFPmut4.R18 | 432 |
| 8 | GFP110A.F35 | GFP165.R20 | 181 |
| 9 | GFP165F.F35 | GFP201.R20 | 124 |
| 10 | GFP201L.F35 | pBADGFPmut4.R18 | 159 |
| 11 | pET16bMMLVmut.F20 | MMLV69.R20 | 228 |
| 12 | MMLV69E.F35 | MMLV147.R20 | 250 |
| 13 | MMLV147Q.F35 | MMLV225.R20 | 250 |
| 14 | MMLV225P.F35 | MMLV313.R20 | 280 |
| 15 | MMLV313W.F35 | MMLV435.R20 | 382 |
| 16 | MMLV435L.F35 | MMLV454.R20 | 73 |
| 17 | MMLV454N.F35 | pET16bMMLVmut.R20 | 698 |
| 18 | MMLV313W.F35 | MMLV374.R20 | 199 |
| 19 | MMLV374I.F35 | MMLV435.R20 | 199 |
| 20 | MMLV454N.F35 | MMLV524.R20 | 226 |
| 21 | MMLV524D.F35 | MMLV605.R20 | 259 |
| 22 | MMLV605V.F35 | pET16bMMLVmut.R20 | 245 |
| 23 | GFP29X.F35 | GFP66.R20 | 127 |
| 24 | GFP66X.F35 | GFP110.R20 | 148 |
| 25 | GFP110X.F35 | GFP165.R20 | 181 |
| 26 | GFP165X.F35 | GFP201.R20 | 124 |
| 27 | GFP201X.F35 | pBADGFPmut4.R18 | 159 |

**Table S3** Calculated CFU (colony forming units) in each experiment. Transformant colonies on each plate were counted, and the total number of colonies was normalized by amount of vector DNA used to transform cells. EP denotes electroporation.


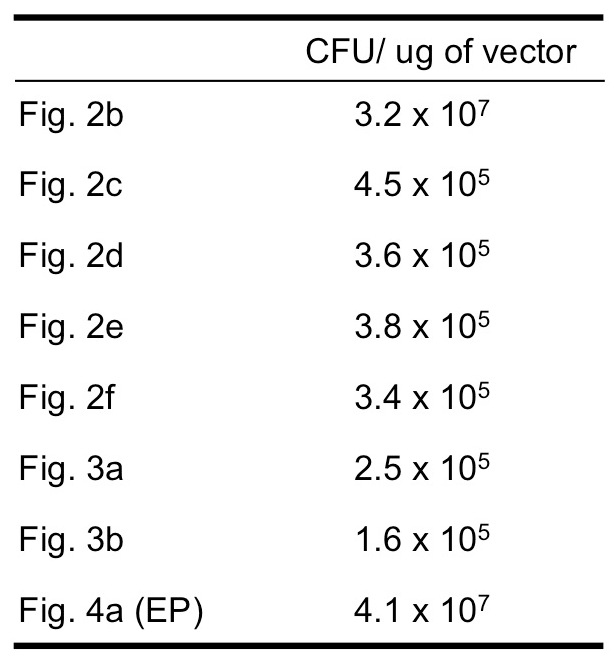


**Table S4.** List of codons at the five randomized sites of GFPuv. Twenty-four plasmids obtained from individual clones in the saturation mutagenesis experiment were sequenced, and the codons of the randomized sites in each mutant are shown.


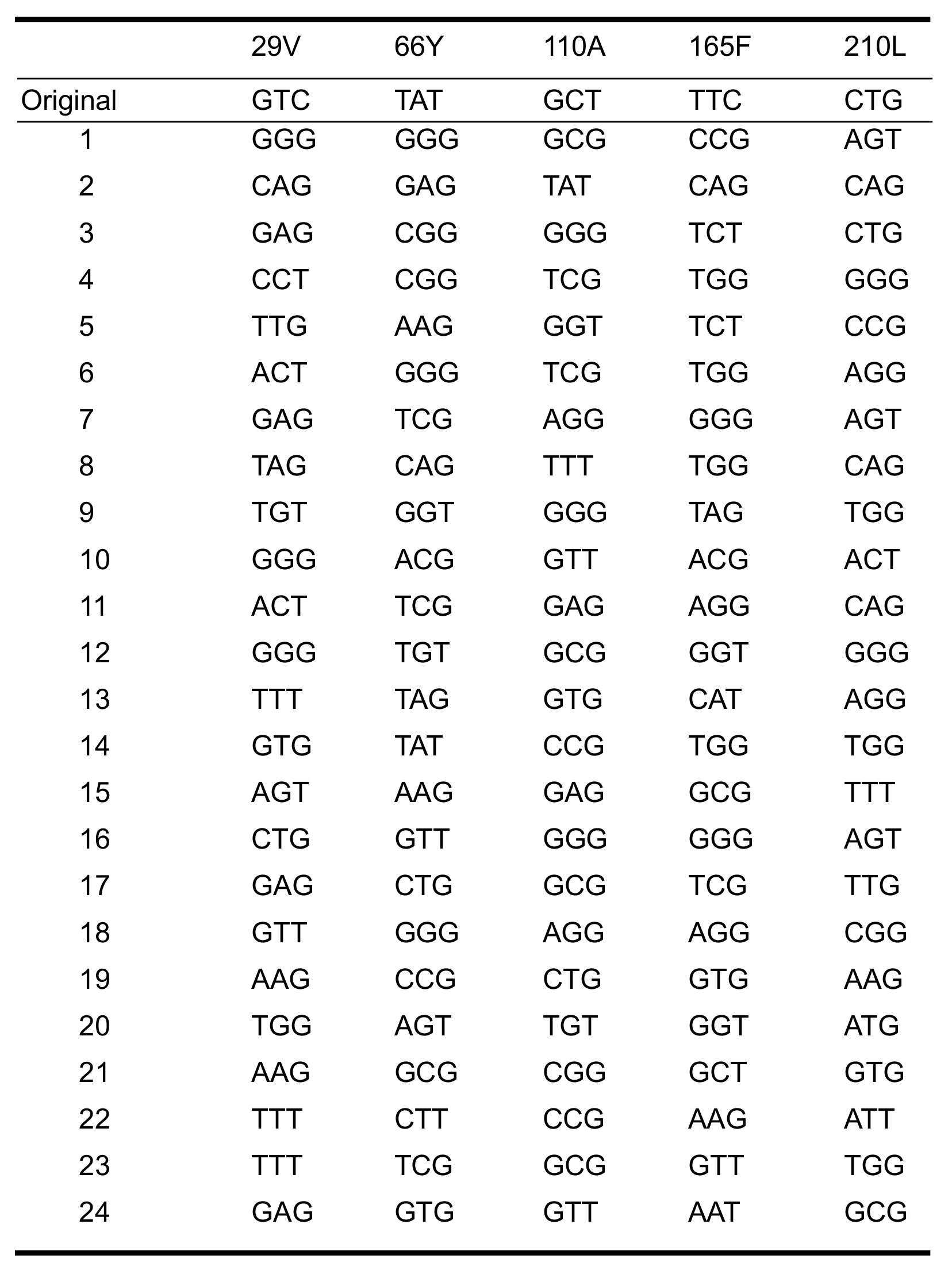


**Table S5.** Number of intragene homologous sequences

|  | Number of pairs | |
| --- | --- | --- |
| Length of homologous pair | MMLV-RT (2019 bp) | GFPuv (720 bp) |
| 10 bp | 1 | 1 |
| 9 bp | 15 | 1 |
| 8 bp | 30 | 0 |
| 7 bp | 128 | 11 |
